# A randomized controlled trial of computerized cognitive training to improve working memory in individuals with elevated repetitive negative thinking: Behavioral and neural outcomes

**DOI:** 10.1016/j.xjmad.2024.100095

**Published:** 2024-12-02

**Authors:** Jessica Bomyea, Morgan M. Caudle, Nathalie Dugas, Raeanne C. Moore, Alan N. Simmons, Michael L. Thomas

**Affiliations:** a Center of Excellence for Stress and Mental Health, VA San Diego Healthcare System, 3350 La Jolla Village Dr, San Diego, CA 92161, USA; b Department of Psychiatry, University of California San Diego, 3120 Biomedical Sciences Wy, La Jolla, CA 92093, USA; c SDSU/UCSD Joint Doctoral Program in Clinical Psychology, 6363 Alvarado Court, Suite 102/103, San Diego, CA 92120–4913, USA; d Department of Psychology, Colorado State University, 201 Behavioral Sciences Building, 1876 Campus Delivery, Fort Collins, CO 80523, USA

**Keywords:** Cognitive training, Working memory, Executive functioning, Repetitive negative thinking, Internalizing disorders

## Abstract

Individuals with mood, anxiety, and traumatic stress disorders frequently experience distressing repetitive negative thinking (RNT) symptoms, which are characterized by pervasive, uncontrollable negative thoughts. Dysfunction in executive functioning processes, particularly the ability to regulate the contents of working memory, are implicated in the development and maintenance of RNT. Guided by the National Institute of Mental Health experimental therapeutics framework, this study aimed to investigate the cognitive effects and effects of two doses of a novel working memory training (WMT) intervention in individuals with elevated RNT across mood, anxiety, and traumatic stress disorders. We conducted a three-arm randomized controlled trial with 8-session and 16-session WMT interventions and a waitlist control group (N = 73). Our primary goal was to establish target engagement of WMT as measured by behavioral and neural indicators of working memory performance. Results revealed that WMT significantly improved working memory performance and resulted in reduced frontoparietal neural activity during a working memory task compared to the waitlist control group, providing evidence of target engagement. Exploratory analyses revealed a potential transfer of training effects to fluid intelligence, a construct related to but distinct from working memory, suggesting broader cognitive benefits of WMT. These data provide evidence that WMT can enhance working memory performance in individuals with RNT. This intervention holds promise as a transdiagnostic approach for ameliorating RNT-related clinical burden, with the 8-session regimen showing pragmatic advantages. Further research is needed to elucidate its impact on clinical symptoms and explore potential cognitive benefits beyond working memory.

## Highlights

• This study investigated a novel working memory training (WMT) intervention.
• WMT resulted in reduced frontoparietal neural activity.
• WMT significantly improved working memory performance.
• WMT may result in transfer of training effects to fluid intelligence.
• This intervention holds promise for ameliorating clinical burden.

## Graphical Abstract

**Figure:**
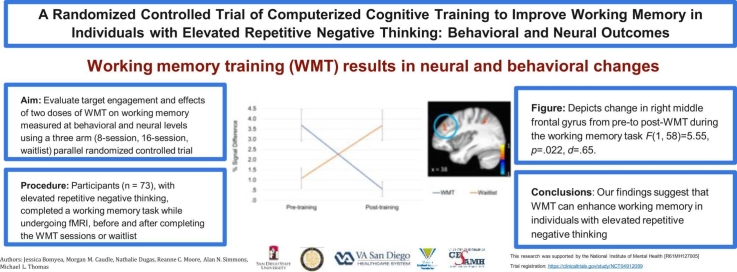

## 1 Introduction

Many individuals with mood, anxiety, and traumatic stress disorders experience repetitive negative thinking symptoms (RNT), defined as frequent, distressing thoughts that are perceived as uncontrollable [1]. Despite differences in the clinical presentation of RNT by disorder (e.g., rumination in depression, worry in anxiety, re-experiencing trauma in PTSD), RNT subtypes load on a single factor reflective of a transdiagnostic, content-independent clinical feature [2], [3], [4]. RNT predicts affective symptoms cross-sectionally [5] and prospectively [6]. Greater pre-treatment severity of RNT has been linked to worse clinical outcomes and greater relapse following evidence-based treatment for depression, anxiety, and PTSD [7], [8], [9], [10] suggesting it may be a pre-treatment marker of non-response [1]. Thus, developing interventions to effectively treat RNT holds promise for ameliorating clinical burden across multiple psychiatric disorders.

RNT is thought to arise from dysfunction of executive control processes responsible for removing irrelevant or unwanted information from working memory, causing individuals to get “stuck” in negative thoughts [11], [12], [13]. Executive functioning includes updating and monitoring information in working memory, inhibiting irrelevant information or behaviors, and shifting between mental activities [14], [15], which are supported by the frontoparietal network (e.g., dorsal anterior cingulate (dACC), dorsolateral prefrontal cortex (dlPFC), inferior frontal gyrus (IFG) [16], [17], [18] and its connection with subcortical and posterior regions[19]. Disorders characterized by elevated RNT demonstrate diminished executive functioning task performance [20], [21], [22], [23], [24], [25], [26], [27] and dysfunction in frontoparietal circuitry [28], [29], [30], [31], [32]. Severity of RNT subtypes (worry [33], [34], rumination [35], [36], re-experiencing symptoms [37], [38]) and transdiagnostic RNT [39], [40], [41] are associated with behavioral and neural indicators of executive functioning. In line with the Research Domain Criteria initiative (RDoC), which focuses on identifying constructs that undergird transdiagnostic clinical complaints [42], executive functioning deficits may be a target for improving RNT symptoms that are pervasive in mood, anxiety, and traumatic stress disorders.

Computerized training interventions providing practice with neuropsychological exercises may improve cognitive function in clinical disorders via experience-dependent neuroplasticity[43]. For example, training targeting executive functioning shows promise for improving cognitive function in individuals with depression[44], anxiety[45], [46], and PTSD[47], [48] and may have beneficial effects on mood and anxiety symptoms[49], [50], [51]. Although to our knowledge no published studies have attempted executive functioning training specifically for transdiagnostic RNT, effects of cognitive training on outcomes like rumination have been mixed; some studies show evidence that training reduces symptoms[51], [52], [53], [54], [55], [56], [57] while others do not[58], [59], [60], [61]. Notably, studies that enrolled individuals based on elevated RNT (i.e., high worriers or high ruminators[55], [56], [58], [59], [60]) did not find evidence that the intervention changed cognitive performance, though individual differences analyses showed that change in cognitive performance was associated with change in RNT. Improved cognitive functioning is an important prerequisite of clinical change in the mechanistic model linking executive functioning deficits to RNT. There remains a need to establish training parameters (e.g., type of training task administered, amount prescribed, and amount of adherence) that are likely to shift cognitive performance in individuals with RNT.

An optimized training program, if designed as a treatment for clinical symptoms, would also manipulate the specific executive functioning component(s) that are most proximal to RNT rather than broad thinking skills. A recent meta-analysis demonstrated that inability to engage working memory interference control – i.e., preventing outdated or irrelevant information from impacting working memory - was the executive function component most robustly associated with RNT[62]. Yet working memory interference control is not isolated by previously studied cognitive training programs such as N-back paradigms (which tap a different working memory subcomponent) or multi-component trainings (CogMed). To address this gap, we previously developed a training program based on a working memory capacity paradigm, which has been shown to rely on interference control ability[63], [64], [65], that was modified to manipulate working memory interference demand. In a series of two pilot studies, WMT with high interference demand resulted in improved cognitive performance as well as improved thought regulation ability in healthy individuals[66] and reduced re-experiencing in individuals with PTSD[48], relative to a sham procedure that contained similar working memory storage demands but low interference demand. These data suggest that targeted training of working memory interference demand could be beneficial for reducing RNT. Determining the cognitive effects of this WMT intervention in a transdiagnostic sample with elevated RNT is a critical next step for evaluating its viability as an intervention.

The objective of the current study was to determine the cognitive effects and examine differences between two doses of WMT (8 versus 16 sessions) in individuals with mood, anxiety, or traumatic stress disorders and elevated RNT. The study design was guided by the National Institute of Mental Health experimental therapeutics framework[67] and R61/R33 grant funding mechanism, which aims to first provide evidence of *target engagement* (i.e., an intervention modulates a pre-specified, modifiable mechanism that underlies the clinical pathology of interest) prior to efficacy studies powered to test the impact of the intervention on clinical symptoms. We evaluated target engagement and effects of two doses of WMT on working memory measured at behavioral and neural levels using a three arm (8-session, 16-session, waitlist) parallel randomized controlled trial [preregistration can be found at: NCT04912089]. Primary analyses focused on the effect of training (both doses combined) as compared to waitlist. We hypothesized that WMT, relative to waitlist, would result in significant improvement in behavioral performance on a working memory task with novel stimuli. Relative effects of the two doses administered (using an a priori-defined effect size benchmark) and attrition rates were examined to determine whether more training sessions resulted in meaningfully larger improvements in working memory performance that would justify the more intensive treatment. The behavioral working memory performance criterion was used as our a priori defined “go” criterion for determining success of target engagement and if WMT should continue to be pursued as a clinical intervention because it was the most direct, proximal indicator of training effects (versus transfer effects to different tasks or neural versus behavioral levels of analysis) and thus likely most sensitive to potential effects and dose differences. As a secondary marker of WMT efficacy observed at the neural level, we evaluated the extent to which WMT modified neural activity during a working memory task. Research suggests that WMT modulates brain activity in frontoparietal regions, though directions of effects have been mixed[68]. Based on our neural data from single-session training[69], we hypothesized that WMT would increase functional activation in frontoparietal regions relative to waitlist. As an exploratory outcome, we examined the extent to which cognitive effects would transfer to other similar skills. Transfer was measured with a composite measure of fluid intelligence - a construct related to but partially separable from working memory[70] – and it was hypothesized that WMT, relative to waitlist, would increase performance.

## 2 Materials and methods

### 2.1 Participants

Participants (N = 73) were enrolled between October 2021 and May 2023 from community settings. All participants met diagnostic criteria for one or more mood, anxiety or traumatic stress disorders, and had a score above the clinical cutoff (32 +) on the Repetitive Negative Thinking Questionnaire-10 (RTQ-10[71]) (see supplementary materials (S2) for full eligibility criteria). Inclusion criteria were evaluated using the Mini International Neuropsychiatric Interview for DSM-5 (MINI Version 7.0.2) and the PhenX Anxiety Disorders Screener (Composite International Diagnostic Interview Screening Scales (CIDI-SC[72]). See Table 1 for demographic and clinical characteristics. Participants provided written informed consent and the protocol was approved by the UC San Diego Institutional Review Board. The study design, a priori-defined primary (cognitive performance), secondary (neural activity as measured with MRI), and exploratory endpoints, as well as the associated statistical analysis plan were reviewed by an external grant scientific review group and confirmed by an independent external data monitor prior to enrollment commencement (who also reviewed study progress annually thereafter). The trial design and associated clinicaltrials.gov registration was updated on two occasions; the first was to accommodate additional symptom inventories to conduct exploratory analyses of effect sizes for clinical effects of training (i.e., to provide effect size estimates of RNT changes for the second phase of the study to be conducted in the subsequent grant award period), and the second was to adjust the total enrollment goal to achieve the desired final sample size (due to a higher than expected number of participants deemed ineligible at the screening visit).

**Table 1:** Baseline Sociodemographic and Clinical Characteristics by Group.

Characteristic	WL M (SD)	WMT M (SD)	8-session subgroup M (SD)	16-session subgroup M (SD)	Statistic (WL versus WMT)
Sex n (% Female)	21(87.5)	35(74.5)	18 (75.0)	17 (73.9)	χ2(1) = 0.16, *p* = 0.40
Age (years)	31.59(7.79)	32.45(8.42)			*F*(1, 69) = 0.17, *p* = 0.68
Education (years)					χ2(6) = 0.28, *p* = 0.35
High School Diploma/GED	0(0)	2(4.3)	0(0)	2(8.7)	
Some post high school, no degree	3(12.5)	6(12.8)	5(20.8)	1(4.3)	
Technical school certificate/degree	2(8.3)	0(0)	0(0)	0(0)	
2-year college degree	2(8.3)	3(6.4)	2(8.3)	1(4.3)	
4-year college degree	12(50)	21(44.7)	10(41.7)	11(47.8)	
Graduate or professional study	5(20.8)	14(29.8)	7(29.2)	7(30.4)	
Race n (%)					χ2(4) = 0.11, *p* = 0.93
Asian	3(12.5)	6(12.8)	5(20.8)	1(4.3)	
Black	0(0)	1(2.1)	1(4.2)	0(0)	
More than one Race	5(20.8)	7(14.9)	5(20.8)	2(8.7)	
White	12(50)	28(61)	13(54.2)	16(69.6)	
Unknown/declined	2(8.3)	4(8.5)	0(0)	4(17.4)	
Hispanic ethnicity n (%)	7(29.2)	15(31.9)	8(33.3)	7(30.4)	χ2(1) = 0.06, *p* = 0.81
Diagnosis Current depression Panic Disorder Agoraphobia Social Anxiety GAD PTSD Specific Phobia	5(20) 3(13) 1(4) 4(17) 9(38) 4(17) 2(8)	12(26) 2(4) 0(0) 10(21) 17(36) 9(19) 3(6)	7(30) 1(4) 0(0) 4(17) 8(33) 4(17) 1(4)	5(22) 1(4) 0(0) 6(26) 9(39) 5(22) 2(9)	χ2(1) = 0.19, *p* = 0.66 χ2(1) = 1.65, *p* = 0.20 χ2(1) = 1.99, *p* = 0.16 χ2(1) = 0.219, *p* = 0.64 χ2(1) = 0.01, *p* = 0.91 χ2(1) = 0.07, *p* = 0.79 χ2(1) = 0.09, *p* = 0.76
RNT−10	40.54(4.67)	39.78(4.38)	40.26(4.38)	39.30(4.42)	*F*(1, 69) = 0.45, *p* = 0.50
QIDS	11.5(4.39)	11.17(4.53)	11.58 (4.84)	10.74(4.26)	*F*(1, 69) = 0.09, *p* = 0.77
GAD−7	8.46(4.63)	9.4(5.54)	9.25(5.46)	9.56(5.74)	*F*(1, 69) = 0.51, *p* = 0.48
SSRI Use n (%)	7(29.2)	9(19.1)	2(8.3)	7(30.4)	χ2(1) = 0.11, *p* = 0.34

*Note*. WL = waitlist, WMT = working memory training, RNT-10 = Repetitive Thinking Questionnaire, QIDS = Quick Inventory of Depressive Symptomatology, GAD-7 = Generalized Anxiety Disorder-7.

### 2.2 Outcome measures

#### 2.2.1 Primary outcome – operation span task (Ospan)

The computerized automated Ospan task is a measure of working memory capacity[73]. This measure was selected as the primary outcome for working memory capacity improvement because it reflects the same level of analysis as the training task (behavioral performance) and used a novel stimuli set (i.e., a different stimuli set than was used in training, to evaluate near transfer). Further details on the Ospan task are in the supplementary materials (S3).

#### 2.2.2 Secondary outcome – MRI reading span task (Rspan)

The computerized automated Rspan task is an additional working memory capacity task previously shown to probe frontoparietal circuitry[74]. Neural data was considered secondary given that it reflects transfer to a novel level of analysis compared to training. Details on the Rspan task are reported in the Supplementary Materials (S3).

#### 2.2.3 Exploratory cognitive generalization outcome: NIH toolbox cognition battery fluid intelligence composite

The NIH Toolbox Cognition Battery version 1.0 (www.nihtoolbox.org) was used to assess cognitive performance on executive function, language, processing speed, working memory, and attention. Performance was summarized using the composite demographically corrected T-scores of crystallized intelligence provided by the tests via the Assessment Center (http://assessmentcenter.net/) (see Supplementary Materials (S3) for further information).

### 2.3 Treatment

#### 2.3.1 Working memory training

The WMT was a modified version of the Rspan task described above. Participants completed three blocks of training in each session, with sessions lasting 30–45 min total. Within each block, participants trained on span sizes of two to six, with three repetitions of each span size presented in random order such that during the three blocks the participant completed 54 trials total. Items and sentences used in the training task derived from four sets (one for each week), and each of the weekly sets was distinct from the stimuli used in the assessment version of the task. Participants were randomly assigned to complete either 2 sessions per week for 4 weeks (8 session condition) based on our prior work[48] or 4 sessions per week for 4 weeks (16 session condition) based on guidance from earlier work advising up to 8 h of training[75]. Training was administered as a telehealth appointment where participants virtually remoted into the task with study staff who oversaw task completion.

#### 2.3.2 Waitlist (WL)

Waitlist participants completed baseline, mid- (week 2), and post-training assessments (week 5). They were offered WMT following the post-training assessment (5 participants initiated; outcome from this phase not included in analyses).

### 2.4 Procedure

Participants completed an intake appointment where they provided consent and initial clinical interviews and self-report measures. Those meeting inclusion criteria completed a separate baseline assessment visit that included cognitive testing and functional magnetic resonance imaging (fMRI). Following completion of this visit, participants were randomized 1:1:1 to WMT (8 or 16 sessions) or WL. Randomization followed a permuted block design and stratified by primary diagnosis (anxiety/PTSD or depression) and age (dichotomized <30 or >=30). Treatment assignment was blinded for the PI but not participants or those administering the training. After the training phase, participants completed two post-training assessment visits where they completed interview, questionnaire, cognitive testing, and fMRI assessments again.

### 2.5 Statistical analyses

#### 2.5.1 Analysis of primary working memory target engagement outcome

Improvements in our proximal indicator of working memory capacity, Ospan behavioral performance, were analyzed using a mixed between-within analysis of variance (ANOVA). Independent variables included treatment group (WMT vs. WL), visit (pre, post), and the interaction of group-by-visit. Analyses were conducted using SPSS version 24 and R version 3.6.1 (https://www.r-project.org/). We conducted a supplemental ANOVA on changes in Rspan performance using behavioral data that was collected during the scan as an exploratory analysis, though neural data was our planned secondary outcome using this paradigm. Data on RNT severity were collected to examine pilot estimates of effect sizes on clinical symptoms within groups; because we did not have planned inferential statistical models they are reported in the Supplemental Materials (S1).

#### 2.5.2 Analysis of dose effect using effect size computation

To compare dose effects, Cohen’s d effect size estimates were compared (8 vs. 16 sessions) for differences in mean change pre- to post-training. We set an a priori benchmark of d > .39 to decide whether an 8 or 16 session version of the task should be used in future studies. Effect sizes for change on primary and secondary outcomes are reported throughout and were calculated using Cohen’s d differences for change scores pre- to post-training.

#### 2.5.3 fMRI analyses

Details of the fMRI analyses, including acquisition parameters and single subject analyses, are reported in the Supplemental Materials (S4).

#### 2.5.4 Sample size determination

Required sample size was determined via power calculations for the primary outcome (Ospan performance). Calculations using R *WebPower* software assuming a two-sided test with alpha set to 0.05, test-retest reliability of.77 (based on our pilot data estimate and consistent with prior published estimates of.77–.80[76]), and attrition of 15 %, indicated a planned sample initial size of n = 65 participants could detect a medium-large effect size (η_p_^2^ =.11–.19) with > 80 % power.

## 3 Results

### 3.1 Preliminary analyses

**Fig. 1:**
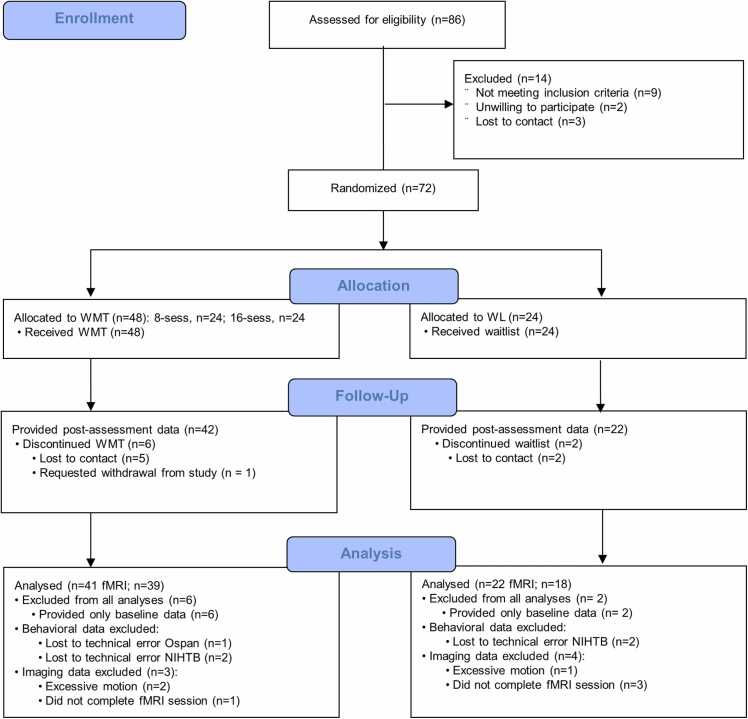
CONSORT flow diagram summarizing participants’ progress throughout the study.

Fig. 1 (CONSORT Diagram) summarizes the flow of participants through the study. There were no statistically significant differences between the groups on baseline clinical characteristics (*p*s > .18; Table 1). There were no statistically significant differences between the groups on baseline Ospan, t(68) = .62, p = .54, or Rspan performance, t(66) = .87, p = .39, or NIH Toolbox fluid intelligence scores, t(68) = .89, p = .38 (descriptives in Table 2). Completion rates were 100 % in the 8-session WMT, 79 % in the 16-session WMT, and 92 % in the WL group; all participants who completed post-training assessments completed the full course of the assigned trainings. As a provisional test of the extent to which training improved performance, we examined change in behavioral performance during the training tasks by week. There was not a significant effect of dose (8 versus 16 session) on training task performance *F*(3117)= 2.58, *p =* .12, ɳ_p_^2^ = .061 but a significant improvement on training task performance over time overall, *F*(3120)= 8.83, *p =* .005, ɳ_p_^2^ = .328.

**Table 2:** Results from the analysis of variance for Group (Waitlist vs. WMT) by Time (pre-, post-training) effects for behavioral indicators across the Ospan, Rspan, and NIH Toolbox measures.

Measure		Baseline *M (SD)*	Post-training Assessment *M (SD)*	Change *M (SD)*	ANOVA Results (Group x Time interaction)			
a. Ospan score (total correct)					F(1,61) = 4.42, *p* = 0.040, d = .56			
	WL	61.00(12.54)	62.86(15.06)	0.27(10.58)				
	WMT	58.98(13.09)	63.57(12.64)	6.27(10.90)				
b. Rspan score (total correct)					F(1,58) = 5.55, *p* = 0.022, d = .65			
	WL	69.30(12.59)	66.71(11.04)	1.58(8.34)				
	WMT	72.68(12.82)	74.10(8.12)	7.80(10.01)				
c. NIH Toolbox (Fluid Intelligence T-score)					F(1,57) = 5.03, *p* = 0.029, d = .63			
	WL	51.96(13.28)	54.74(13.21)	1.36(7.66)				
	WMT	49.49(9.55)	54.90(9.06)	5.88(6.99)				

*Note*. WL = waitlist, WMT = working memory training, means reported for all data regardless of completer status

### 3.2 Primary Target Engagement: Ospan Behavioral Performance

Results for the effect of training on Ospan performance are presented in Table 2a. Results revealed a significant group (WMT vs. WL) by time effect on Ospan performance, *F*(1,61)= 4.42, *p* = .040, *d*= 0.56.^2^ Results of follow up paired t-tests revealed that individuals receiving WMT showed significant improvements in Ospan scores, *t*(40) = 3.68, *p* = .001, *d*= .57, while those in WL did not, *t(*21)= 0.12, *p* = .91, *d*= .03. Examination of effect size of mean change between WMT groups on the Ospan score (*d*=.30) did not exceed the a priori-defined clinically meaningful difference (d>.39) between the 8 versus 16-session groups (8-session WMT: *M*=4.92(8.07); 16-session WMT: *M*= 8.18(14.03)).

### 3.3 Secondary target engagement: rspan neural activation and behavioral performance

**Fig. 2:**
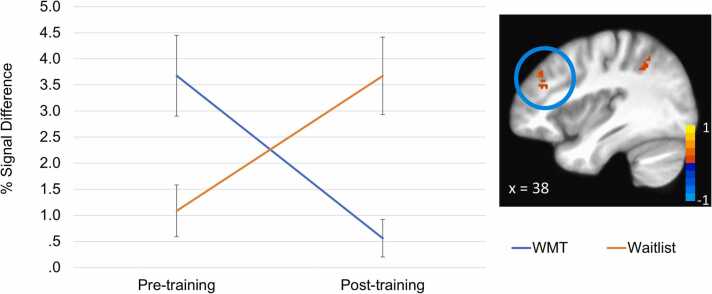
Change in right middle frontal gyrus (x = 38, y = 37, z = 32) from pre- to post-training during the encoding phase of the R-Span task. Data reflects parameter estimates (left) extracted from the largest cluster emerging from the Group x Time linear mixed effects model (voxel-wise a priori probability of.001 with corrected cluster-wise activation probability of.05 within the working memory mask). Note. WMT = Working Memory Training, WL = waitlist.

Results from the linear mixed effects analysis are presented in Table 3. WMT participants displayed significant reductions in neural activation from pre- to post-training across several frontoparietal regions, including the bilateral middle frontal gyri (MFG), right inferior parietal lobule and right superior parietal lobule compared to WL. Group differences over time in the largest cluster (right MFG) are illustrated in Fig. 2. Behavioral data from the fMRI task revealed that individuals in the WMT group demonstrated greater improvement in total correct relative to those in WL, *F*(1, 58)= 5.55, *p* = .022, *d*= .65 (Table 2b). Results of follow up paired t-tests revealed that individuals receiving WMT showed significant improvements in Rspan scores, *t*(40) = −4.99, *p* < .001, *d*= .78, while those in WL did not, *t(*18)= −0.83, *p* = .42, *d*= .19. On a post-hoc exploratory basis we examined Spearman correlations between neural activation (average change in the largest four ROIs (>20voxels) and average change in span task performance). Results revealed that greater increases in working memory performance were associated with greater decreases in neural activation in frontoparietal regions, *r* = −.34, *p* = .009.

**Table 3:** Results from the linear mixed effects analysis of Group (Waitlist vs. WMT) by Time (pre-, post-training) on percent signal change for the Encoding phase of the R-Span Task.

ROI	Voxels	x	y	z	t-stat	Region	BA	Mean Change (SD)	
								WL	WMT
1	51	38	37	32	4.21	Right Middle Frontal Gyrus	9	2.46(3.2)	−2.56(5.09)
2	41	38	−51	46	4.10	Right Inferior Parietal Lobule	40	1.47(3.3)	−2.04(3.93)
3	34	−38	31	32	4.27	Left Middle Frontal Gyrus	9	1.96(1.98)	−1.78(3.07)
4	24	−47	34	32	4.40	Left Middle Frontal Gyrus	46	4.58(4.53)	−6.24(12.37)
5	18	11	−74	58	4.48	Right Superior Parietal Lobule	7	3.63(9.3)	−8.81(18.92)
6	17	0	14	45	3.99	Right Cingulate Gyrus	32	3.35(5.41)	−3.05(5.94)
7	17	22	−2	53	4.49	Right Medial Frontal Gyrus	6	1.84(2.45)	−1.90(2.78)
8	16	−3	15	53	4.06	Left Superior Frontal Gyrus	8	2.18(5.01)	−3.51(6.86)
9	16	−6	3	56	4.22	Left Medial Frontal Gyrus	6	2.64(3.03)	−1.91(5.35)
10	15	27	−60	−27	4.30	Right Declive		2.29(3.35)	−1.97(4.37)
11	15	−25	9	58	3.84	Left Superior Frontal Gyrus	6	0.82(2.14)	−2.41(5.37)
12	14	9	20	45	3.94	Right Cingulate Gyrus	32	0.86(2.56)	−2.15(4.08)
13	14	−48	−43	48	3.97	Left Inferior Parietal Lobule	40	0.90(2.48)	−1.78(3.37)
14	13	−38	−2	39	4.24	Left Precentral Gyrus	6	0.87(2.33)	−1.67(2.3)
15	12	32	16	48	3.94	Right Middle Frontal Gyrus	8	0.49(2.49)	−1.63(3.55)
16	12	26	−2	60	4.31	Right Middle Frontal Gyrus	6	2.63(6.43)	−2.11(4.02)
17	11	−34	−65	−33	4.50	Left Uvula		0.94(5.52)	−2.32(6.05)
18	10	44	−40	43	4.05	Right Inferior Parietal Lobule	40	1.32(2.49)	−2.0(3.91)

*Note.* Change reflects the difference from baseline (post-training activation minus pre-treatment activation). WL = waitlist, WMT = Working Memory Training.

### 3.4 Exploratory target engagement: NIH toolbox behavioral performance

Results for the effect of training on NIH Toolbox Fluid Intelligence scores are presented in Table 2c. Results revealed a significant group by time effect on scores, *F*(1,57)= 5.03, *p* = .029, *d*= 0.63. Results of follow up paired t-tests revealed that individuals receiving WMT showed significant improvements in scores, *t*(39) = 5.32, *p* < .001, *d*= .83, while those in WL did not, *t*(18) = 0.78, *p* = .45, *d*= .18.

## 4 Discussion

The present study sought to evaluate evidence of target engagement in participants completing WMT relative to WL as an initial step toward evaluating this intervention within an experimental therapeutics framework. Results indicated that WMT resulted in greater improvements in working memory capacity performance than WL. This effect was observed on the Ospan task, which was designated as the primary outcome for determining that the intervention engaged the working memory target as hypothesized. Moreover, the WMT group demonstrated reductions in neural activity in frontoparietal regions during a working memory capacity task relative to WL. These data suggest that the conditions differentially engaged working memory based on both neural and fMRI task data. A secondary question in our study was the extent to which greater dose, operationalized as a larger number of treatment sessions over the 4-week intervention period, would result in meaningfully better target engagement as measured by working memory capacity performance. The pattern of means in the 16- versus 8-session groups suggested that longer treatment may have conferred greater benefit. However, the size of this effect was slightly below our pre-determined effect size threshold for determining if the more intensive treatment was warranted. Finally, effects of training were observed on a broader composite measure of fluid intelligence.

Results add to a growing literature demonstrating beneficial effects of cognitive training on cognitive performance and extend these findings to a transdiagnostic, clinical sample of individuals with elevated RNT. Earlier work showed that cognitive training can enhance neuropsychological performance, though effective training methods for improving cognitive performance in those with elevated RNT have been elusive. This study provides evidence that the WMT exercises successfully shifted performance on a working memory capacity task known to tap interference control ability – the executive functioning component thought to be most proximal to RNT [62]. Individuals who received WMT also showed improvements relative to WL on a composite of fluid intelligence, which contains components of working memory but encompasses a broader set of thinking skills. Our findings align with prior work showing that effects of cognitive training can generalize to broader constructs like fluid intelligence [77] and neural activation [68], and align with prior positive trials of training using working memory capacity paradigms in psychiatric populations [47], [48]. Notably, critical reviews of cognitive training paradigms have highlighted equivocal effects in showing transfer of gains beyond trained tasks [78], which is particularly important if a training intervention ultimately aims to shift clinical outcomes. Substantial differences between methodological features of the current study and those included in recent meta-analyses of WMT make direct comparisons difficult. For example, studies included in prior meta-analyses primarily tested training paradigms like N-back and CogMed, which tap dissociable cognitive subconstructs from a specific working memory capacity training [79], and enrolled children and adults were either healthy or diagnosed with learning disabilities and thus not necessarily reflective of individuals with internalizing disorders. Thus, future work will be needed to determine the extent of generalization of training and the replicability of effects in this population.

WMT also resulted in reduction in neural activity in regions of the frontoparietal network while engaging in a working memory capacity task. We hypothesized that WMT would result in increased neural activity during working memory task performance based on our preliminary data using single-session training [80]. However, results revealed an opposite pattern whereby those in the WMT group showed relative decreases in frontoparietal neural activation over time relative to those in the WL group. While somewhat unexpected, direction of neural training effects vary in published literature –some studies report increased activation, but an increasing majority report decreased neural activity which is interpreted as improved neural efficiency [68], [81]. The relative timing of neural data collection could partially account for discrepant findings, with increased activation observed during short training (or early in a sequence of trainings [82]) and decreased activation in longer, multi-session formats [83], [84]. Findings are consistent with theoretical models suggesting impaired neural efficiency in anxiety and links between rumination and increased neural activation in depression (i.e., greater load demands couple with poor efficiency leading to increased neural activity [85], or greater neural activity may be reflective of individuals dually managing RNT and task demands [86], [87]) that may ameliorate with treatment. While future work will be needed to evaluate the parameters leading to increased versus decreased activation, observed differential effects across groups in the current study across behavioral and neural assessments supports transfer of training effects across units of analysis.

More training intuitively seems likely to produce greater cognitive improvement. However, differences in the effect size between 8 and 16 session training programs were relatively modest, particularly regarding the amount of additional time in the longer treatment. This finding converges with several large-scale meta-analyses of cognitive training paradigms showing that effect sizes are similar in high versus low doses of training [68], [88], [89]. One possible explanation for the observed similarities in working memory improvements across groups could be that both high and low doses generate sufficient cognitive enhancement to engage neuroplasticity, with minimal improvement seen beyond this level. Higher fatigue or boredom could also occur in those completing more sessions, leading to truncated performance improvements. Notably, training dose was manipulated within the same time frame for each group so we were unable to examine spacing effects [90]. Future work is needed to determine if a longer duration could result in different outcomes, as well as whether individual variability in baseline cognitive function or clinical features would make one dose relatively more optimal for a given person. Attrition was somewhat higher in the 16-session group, suggesting greater feasibility of shorter training. Selection of dose should ideally balance efficacy with other elements of treatment including complexity and patient burden [91]. Together, the similarity in outcomes observed across groups combined with the greater time required in 16 sessions provisionally suggest that a higher dose as operationalized in this trial may not be warranted.

Several study limitations should be considered in interpreting findings. First, a waitlist comparator was used in the trial because the study was designed to be a preliminary test of potential signal for shifting working memory function (versus a clinical efficacy study). We considered both an open trial design and a sham training comparator to enhance statistical power and experimental rigor, respectively. We opted to include a repeated assessment control waitlist design because it balances control of key variables (time, assessment task practice) with feasibility within the short time duration associated with the funding mechanism that supported this trial. Future work is needed with an active comparator (e.g., sham training that controls for non-interference control demands of cognitive tasks; e.g., [48]) to rule out potential general placebo effects, specificity of the intervention to the desired working memory intervention versus generic effects of cognitive activity with the computer interface, etcetera on clinical and target engagement effects. The study was designed and registered to test the effects of training on working memory outcomes; the specific goals of the supporting grant proposal included tests of near (primary) and far (secondary) transfer on cognitive outcomes. Clinical outcomes were considered exploratory outcomes to inform the planned subsequent trial, and as such we did not include a priori aims or sufficient power to robustly evaluate clinical efficacy. Prior data show that clinical symptom effects are more modest than near transfer effects on cognitive tasks [44], so a larger-scale clinical trial is needed to adequately evaluate the relationship between cognitive change and change on RNT and other clinical outcomes and test the bounds of transfer beyond the working memory capacity, neuroimaging, and fluid intelligence metrics in this study. While the use of a transdiagnostic sample with a shared clinical presentation characterized by RNT is a strength and aligns with RDoC-based models, it precludes drawing conclusions about effects within the specific disorders included. Finally, we were under-powered to examine the extent to which baseline clinical and cognitive characteristics moderated outcomes. Given data suggesting that some cognitive training interventions may be relatively more beneficial for those with initial deficits [92], inclusion of these analyses within the context of larger clinical trials will be important.

### 4.1 Conclusion

In conclusion, this experimental therapeutics clinical trial advances our understanding of WMT as a potential intervention for individuals suffering from mood, anxiety, or traumatic stress disorders with elevated RNT. Working memory deficits characterize these disorders and are thought to contribute to the etiology of persistent RNT, suggesting they may be a beneficial treatment target. The observed engagement of the clinical target, working memory capacity as measured behaviorally and neurally, and links to clinical change underscore the potential therapeutic benefits and mechanism of action of this cognitive intervention. Moreover, the modest dose-dependent effect challenges existing assumptions about the optimal amount of WMT. Data provide initial support for the use of this intervention in future research efforts in individuals with mood, anxiety, or traumatic stress disorders. Future directions include replication and extension to larger-scale, well-controlled clinical trials.

## Funding

This research was supported by the 10.13039/100000025National Institute of Mental Health [R61MH127005].

Trial registration: https://clinicaltrials.gov/study/NCT04912089.

## Declaration of Competing Interest

The authors declare the following financial interests/personal relationships which may be considered as potential competing interests: Reanne C. Moore reports a relationship with KeyWise Inc. that includes: equity or stocks. Reanne C. Moore reports a relationship with NeuroUX Inc. that includes: equity or stocks. Dr. Raeanne C. Moore has a relationship with and a co-confounder of KeyWise, Inc. and NeuroUX Inc. If there are other authors, they declare that they have no known competing financial interests or personal relationships that could have appeared to influence the work reported in this paper.

## Footnotes

Appendix A Supplementary data associated with this article can be found in the online version at doi:10.1016/j.xjmad.2024.100095.

## Footnotes

2 An intent to treat analyses was included post-hoc to supplement the pre-planned analyses as stated in the grant proposal. This analysis resulted in the same pattern of findings and statistical significance for the primary outcome, such that WMT outperformed WL, *F*(1.69)= 4.08, *p* < .05, and thus only pre-planned analyses are reported here.
