# Supplementary material for: A randomized controlled trial of computerized cognitive training to improve working memory in individuals with elevated repetitive negative thinking: Behavioral and neural outcomes
Source: J Mood Anxiety Disord. 2024 Dec 2;9:100095. doi: 10.1016/j.xjmad.2024.100095 (PMC12244123; doi:10.1016/j.xjmad.2024.100095)
Supplement: Supplementary file 1 — Supplementary material [file mmc1.docx]

**A Randomized Controlled Trial of Computerized Cognitive Training to Improve Working Memory in Individuals with Elevated Repetitive Negative Thinking: Behavioral and Neural Outcomes**

**Supplemental Materials**

**S1. Methods: Exploratory** **clinical generalization outcome – repetitive negative thinking severity.**

At baseline, mid-training, and post-training, participants completed a battery of self-report questionnaires assessing types of RNT, including the RNT-10, Perseverative Thinking Questionnaire^1^, Ruminative Response Scale^2^, and Penn State Worry Questionnaire^3^. To reduce the number of multiple comparisons in the absence of an a priori-defined clinical measure, scores were standardized and averaged into a single composite score at each time point (baseline, mid-point, post-training). Changes in RNT over time at pre-, mid-, and post-training were examined using repeated measures ANOVAs with linear and quadratic effects tested to evaluate trends over time. In addition, we examined the relationship between RNT and target engagement by analyzing the extent to which change in our primary outcome (Ospan pre-post change scores) related to RNT contrasts over time across groups.

**S2. Methods: Inclusion and Exclusion Criteria**

Participants were recruited from community settings via posted flyers and digital advertisements. Inclusion criteria were: (1) age 21 to 55, (2) fluent in English, (3) meeting diagnostic criteria for one or more mood, anxiety, or traumatic stress disorders,(4) a score above the clinical cutoff (32+) on the Repetitive Negative Thinking Questionnaire-10 (RTQ-10^4^), (5) outpatient status, and (6) 6-week stability if taking an SSRI. Exclusion criteria were (1) past year diagnosis of severe alcohol use disorder, (2) past year moderate or greater substance use disorder, (3) lifetime history of psychotic or bipolar disorder, (4) acute suicidality necessitating immediate clinical intervention, (5) neurodegenerative or neurodevelopmental disorders, (6) history of moderate or severe traumatic brain injury or other known neurological condition, (7) sensory deficits that would preclude completing tasks, (8) conditions unsafe for completing MRI scanning (e.g., metal in the body; pregnancy), (9) currently receiving psychosocial treatment, and (10) currently receiving psychiatric pharmacotherapy, except SSRIs. Other psychiatric disorders that were not considered exclusionary (e.g., OCD, mild substance use disorder) to maximize generalizability.

**S3. Methods: Primary, secondary, and exploratory outcomes**

**Primary Outcome – Operation Span Task (Ospan)**

Each trial began with a fixation cross presented in the center of the screen for 500ms, followed by a blank screen for 500ms. Next, an unsolved equation (e.g., 1+2=) was presented on the screen. Once a participant indicated they had solved the equation, the next screen appeared. The following screen displayed a number above two boxes labeled ‘True’ or ‘False.’ Participants indicated whether the number presented was the answer to the unsolved equation on the previous screen by selecting the appropriate choice. This was followed by a letter presented on the screen for 800ms, and then another equation. At the end of each set of letters and equations, a recall screen with twelve letters was presented. Participants selected the letters they had seen in serial order of presentation using the mouse. Tested spans ranged from two to seven, each span having three repetitions each. The primary outcome analyzed was total number of correctly recalled letters.

**Secondary Outcome: MRI Reading Span Task (Rspan)**

Participants were instructed to remember items presented while simultaneously solving a secondary processing task in which they decided if a sentence was logically correct. First, a fixation cross was presented on the screen for 500ms, followed by a sentence (e.g., “Jane walks her car in the park”). Once the participant indicated they read the sentence, they were shown a screen with two boxes, one marked “True” and one marked “False”. The participant would select “True” if the sentence was semantically logical and “False” if it was illogical. The following screen showed an item (number or letter) for 500ms, which the participant was instructed to remember. Sets of sentences and items continued until the end of the trial, where the participant was shown a recall screen with twelve items. They were instructed to select the letters in the order presented using a joystick-operated mouse cursor. When the recognition test was complete, the next trial began. Participants received feedback about their sentence accuracy at the end of each trial. During the instructions, participants were told to keep their accuracy at 85% or above. Spans of 2, 4, and 6 were tested for 7 repetitions each. The behavioral outcome was the total number of correctly recalled items.

**Exploratory cognitive generalization outcome: NIH Toolbox Cognition Battery Fluid Intelligence Composite**

The tests were administered by a trained study coordinator using guidelines from the NIH Toolbox Administration. Tests administered included the Dimensional Change Card Sort, Flanker Inhibitory Control and Attention, Picture Vocabulary, Pattern Comparison Processing Speed, Oral Reading Recognition, and List Sorting Working Memory.

**S4. Methods - Imaging parameters and analyses**

**Acquisition parameters.** Participants were scanned in a 3T Siemens Prisma scanner using a 32-channel head array coil. Each scanning session included a three-plane scout scan, a sagittally acquired sequence for acquiring T1-weighted images (MPRAGE) [T1:FOV 240x256cm; matrix: 300x320; slices 208; slice thickness: 0.80mm; TR 2400ms, TE: 2.22ms, flip angle: 8, inversion time 1000ms], and a T2*-weighted axially acquired multi-band echo-planar imaging (EPI) scan to measure blood oxygen level dependent (BOLD) signals during the task [2.0mm x 2.0mm x 2.0mm; 104 x 104 acquisition matrix, TR .8ms, TE 37ms, flip angle 52]. Voxel-wise activation data within an a-priori defined working memory mask using neuroanatomical atlases of regions relevant to working memory (NeuroSynth v5 “working memory” Topic 045) were entered into a linear model (AFNI: 3dLME)^5^ to evaluate differences in activation across groups (WMT vs. WL) over time (pre-, post-training) to the working memory encoding size regressor of interest. AFNI’s updated 3dClustSim^5,6^ program was used to conduct permutation testing to control for multiple corrections within the mask (voxel-wise a priori probability of p<.001, corrected cluster-wise activation probability of 0.05). We then extracted parameter estimates from clusters derived from the group by time interaction effect to plot effects by group. Three participants failed to meet data quality standards (extreme outliers in DVARs (1) or >15% censored data (2)) and were removed from neuroimaging analyses.

**Single subject analyses***.* The data were preprocessed and normalized to MNI coordinates using tools available in ANTsR, a statistical interface between Advanced Normalization Tools Software, R software, and Analysis of Functional NeuroImages (AFNI). fMRI preprocessing steps consisted of removal of temporal outliers (AFNI:3dDespike), field inhomogeneity correction (ANTsR:abpN4), and slice time correction (AFNI:3dTshift). Motion correction and CompCor estimation correction were also included as part of this processing pathway, and motion and CompCor correction regressors were removed as part of the preprocessing steps. Outlying acquisitions (AFNI 3dToutcount) and the first ten time points were censored from the time series. Data were aligned to individual anatomical and MNI template (ANTsR:antsRegistration/antsApplyTransforms) for group comparisons. Response regressors were generated for phases of the task based on the idiographic timing of presentation for each phase for each participant, and included sentence reading and verification, stimuli encoding weighted by set size and interference demand of a given trial, and stimuli recall weighted by set size and interference demand of a given trial^7^.

**S3. Results - Exploratory RNT outcomes:**

Results revealed a trend level quadratic effect of group over time, *F*(1,62)=4.01, *p*=.050, ɳ^2^_p_=.06, with reductions in severity observed over time in the WMT group, *F*(1,41)=4.40, *p*=.042, ɳ^2^_p_=.10 (M_baseline_=.05(.74), M_mid_=-.13(.84), M_post_=-.06(.77)). No statistically significant linear effects were observed in the WMT group, *p*>.35, ɳ^2^_p_=.02. No statistically significant quadratic, *p*=.96, ɳ^2^_p_=.04 or linear, *p*=.35, ɳ^2^_p_<.001 effects were observed in the WL group (M_baseline_=.09(.64), M_mid_=.20(.72), M_post_=.10(.87)). We examined the extent to which change in Ospan performance related to the quadratic effects across groups, which revealed an s interaction of group by time by change in Ospan, *F*(2,60)=3.26, *p*=.045, ɳ^2^_p_=.10. Follow up analyses within groups were conducted to explore directionality. In the WMT group, greater improvement in Ospan was associated with the quadratic trend of RNT reduction but this did not reach statistical significance, *F*(1,39)=3.43, *p*=.072, ɳ^2^_p_=.08. No statistically significant effects were observed in the WL group, *p*s>.27, ɳ^2^_p_=.06.

**S4. Results – Exploratory outcomes: effects of SSRI medication status**

Exploratory analyses were conducted to examine group by time interaction effects, controlling for SSRI medication status, on the span tasks, fluid composite scores, and five largest regions of interest from the neural data. Supplemental table 1 reports the group by time interactions from the original manuscript along with the group by time interactions controlling for SSRI use status. The group by time interaction effects, controlling for SSRI medication status, remained statistically significant for the five largest regions of interest (p < .005). All results remained unchanged.

**S5. Results - Exploratory outcomes: effects of biological sex**

Exploratory analyses were conducted to examine group by time interaction effects, controlling for sex, on the span tasks, fluid composite scores, and five largest regions of interest from the neural data. Supplemental table 2 reports the group by time interactions from the original manuscript along with the group by time interactions controlling for sex. The group by time interaction effects, controlling for sex, remained statistically significant for the five largest regions of interest (p < .05). All results remained unchanged.

**Supplement Table 1**

*Results from the analysis of variance for Group (Waitlist vs. WMT) by Time (pre-, post-training) effects for behavioral indicators across the Ospan, Rspan, and NIH Toolbox measures.*

| **Measure** | **Group x Time Interaction**  **(Controlling for SSRI use status)** | **SSRI use status x Time Interaction** |
| --- | --- | --- |
| Ospan score (total correct) | F(1,60) = 4.44, *p* = 0.039 | F(1,60) = 0.09, *p* = 0.765 |
| Rspan score (total correct) | F(1,57) = 5.79, *p* = 0.019 | F(1,57) = 0.49, *p* = 0.488 |
| NIH Toolbox (Fluid Intelligence T-score) | F(1,56) = 4.66, *p* = .035 | F(1,56) = 0.15, *p* = .697 |

*Note*. WL = waitlist, WMT = working memory training, means reported for all data regardless of completer status

**Supplement Table 2**

*Results from the analysis of variance for Group (Waitlist vs. WMT) by Time (pre-, post-training) effects for behavioral indicators across the Ospan, Rspan, and NIH Toolbox measures.*

| **Measure** | **Group x Time Interaction**  **(Controlling for Sex)** | **Sex x Time Interaction** |
| --- | --- | --- |
| Ospan score (total correct) | F(1,60) = 4.42, *p* = 0.040 | F(1,60) = 0.08, *p* = 0.785 |
| Rspan score (total correct) | F(1,57) = 5.84, *p* = 0.019 | F(1,57) = 1.45, *p* = 0.234 |
| NIH Toolbox (Fluid Intelligence T-score) | F(1,56) = 4.66, *p* = .035 | F(1,56) = 0.89, *p* = .697 |

*Note*. WL = waitlist, WMT = working memory training, means reported for all data regardless of completer status

**References**

1. Ehring T, Zetsche U, Weidacker K, Wahl K, Schonfeld S, Ehlers A. The Perseverative Thinking Questionnaire (PTQ): validation of a content-independent measure of repetitive negative thinking. *J Behav Ther Exp Psychiatry.* 2011;42(2):225-232.

2. Nolen-Hoeksema S, Morrow J. A prospective study of depression and posttraumatic stress symptoms after a natural disaster: the 1989 Loma Prieta Earthquake. *J Pers Soc Psychol.* 1991;61(1):115-121.

3. Meyer TJ, Miller ML, Metzger RL, Borkovec TD. Development and validation of the penn state worry questionnaire. *Behaviour Research and Therapy.* 1990;28(6):487-495.

4. McEvoy PM, Thibodeau MA, Asmundson GJG. Trait Repetitive Negative Thinking: A Brief Transdiagnostic Assessment. *J Exp Psychopathol.* 2014;5(3).

5. Cox RW. AFNI: software for analysis and visualization of functional magnetic resonance neuroimages. *Comput Biomed Res.* 1996;29(3):162-173.

6. Cox RW, Reynolds, R. C., Taylor, P. A. AFNI and clustering: False positive rates redux. *bioRxiv.* 2016.

7. Bomyea J, Taylor CT, Spadoni AD, Simmons AN. Neural mechanisms of interference control in working memory capacity. *Hum Brain Mapp.* 2018;39(2):772-782.
